# Highly effective photon-to-cooling thermal device

**DOI:** 10.1038/s41598-019-55546-4

**Published:** 2019-12-17

**Authors:** Yanpei Tian, Lijuan Qian, Xiaojie Liu, Alok Ghanekar, Gang Xiao, Yi Zheng

**Affiliations:** 1 Department of Mechanical and Industrial Engineering, Northeastern University, Boston, MA 02115 USA; 2 Department of Physics, Brown University, Providence, RI 02912 USA; 3 Artech LLC, Morristown, NJ 07960 USA

**Keywords:** Mechanical engineering, Metamaterials, Solar energy

## Abstract

Photon-to-cooling phenomenon relies on the atmospheric transparency window to dissipate heat from the earth into outer space, which is an energy-saving cooling technique. This work demonstrates a highly effective aluminized Polymethylpentene (PMP) thin-film thermal structure. The emissivity of aluminized PMP thin films matches well to the atmospheric transparency window so as to minimize parasitic heat losses. This photon-to-cooling structure yields a temperature drop of 8.5 K in comparison to the ambient temperature and a corresponding radiative cooling power of 193 W/m2 during a one-day cycle. The easy-to-manufacture feature of an aluminized PMP thin film makes it a practically scalable radiative cooling method.

## Introduction

Cooling technologies are substantial for energy-saving buildings and thermal management applications. Current cooling approaches rely mostly on compressors and fluid circulation systems with complex mechanical apparatus and high energy consumption. Passive cooling systems such as photon-to-cooling technology provides an alternative approach as energy conserving devices owing to their capability to operate without external energy input^1–4^. Radiative cooling depends on the high transparency of earth’s atmosphere at mid-infrared wavelengths (7.8 *μ*m ~ 13 *μ*m)^5,6^ and it corresponds to the selective optical absorption of polymer films in this range, such as Polyvinyl chloride (PVC)^7^, Polyvinyl fluoride (PVF)^8–12^, and Polymethylpentene (PMP), also known as TPX^13,14^. Due to its low cost, high effectiveness and flexibility to industrial productions, polymer films are highly promising materials for photon-to-cooling applications. Though all the emission spectra of the abovementioned polymer films cover the atmospheric transparency window, PMP has a better performance due to its higher emittance near 8 *μ*m^13^. The optical properties of PMP from near-infrared to mid-infrared wavelengths have not been well reported in the literature. It is significant and necessary to investigate optical properties, such as complex refractive indices of PMP free-standing films for residential and commercial radiative cooling applications.

Various methods have been employed for investigating the refractive index of thin films, such as refractometry and ellipsometry^15–17^. Refractometry is an very accurate method to measure the complex refractive index of ultra-thin film samples with a thickness from a few angstroms to several micrometers^18^. However, it loses accuracy for optically thick samples when the phase shift is greater than several 2*π*^19^. Furthermore, ellipsometry is typically conducted in the reflection setup by measuring the change in polarization as the incident light interacts with the materials of interest^20^, and it requires that thin films are deposited on a smooth highly reflective substrate. It is commonly known that this method is not suitable for thin films of tens or hundreds of micrometers that are commercially available as free-standing films. In this work, refitting the reflectance spectrum of thin films with various thicknesses according to Lorentz-Drude has been proven to be a reliable and accurate approach to estimate refractive indices of materials of interest^21–23^.

PMP is a transparent thermoplastic polymer of 4-Methyl-1-pentene repeating units^24^. It has a smallest density of 0.83 g/cm^3^ in all the existing polymers. The transmittance of PMP in the visible region is slightly lower than Poly(methyl methacrylate) (PMMA), which is often used as a lightweight alternative material to glass^25^. The high emissivity of PMP between 7 *μ*m and 13 *μ*m is attributed to its functional groups and their corresponding molecular vibrations under infrared light irradiation. Consequently, when PMP is aluminized (approximately 250 nm thin) on its backside, it displays an extremely high reflectance in both visible and near-infrared regions while it exhibits high emittance matching perfectly with the atmospheric transparency window^13^. These advantageous optical properties with its relatively high mechanical strength^26^ which ensures PMP standout to be applied for scalable photon-to-cooling applications^14^.

In this paper, free-standing PMP thin films of various thicknesses are manufactured using the hot-press technique. For the first time, the complex refractive indices of PMP are determined by refitting the transmittance spectra using the Lorentz-Drude model and reported in near-/mid-infrared regions ranging from 0.9 *μ*m to 20 *μ*m. To validate the predicted refractive indices of PMP thin films, the transmittance and reflectance spectra are calculated using extracted refractive indices and compared with experimental data. The thermal performance of fabricated aluminized PMP radiative coolers is investigated for passive daytime cooling during a 24-hour cycle. A temperature drop of 8.5 K in comparison to ambient temperature and corresponding radiative cooling power of 193 W/m^2^ during a one-day cycle are achieved to validate the high performance of photon-to-cooling thermal structures.

## Results

### Transmittance spectra analysis

**Figure 1:**
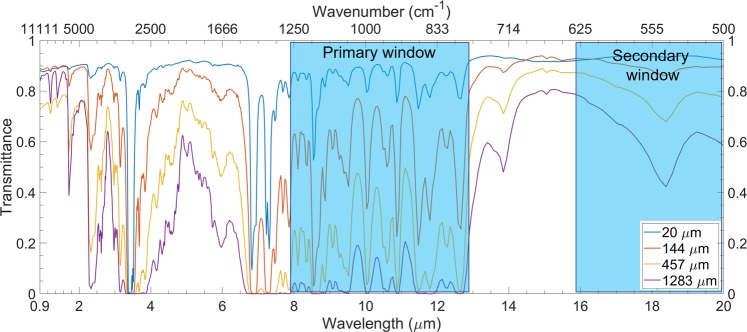
Transmittance measurement of PMP free-standing films with various thicknesses with two atmospheric transparency windows.

Eight samples of PMP free-standing films with thicknesses of 20 *μ*m, 48 *μ*m, 100 *μ*m, 144 *μ*m, 199 *μ*m, 386 *μ*m, 457 *μ*m, and 1283 *μ*m are prepared by the hot-press method. Transmittance spectra of the 1283 *μ*m thick samples show a high absorptance, which match two atmospheric transparency windows (nearly unity absorptance in the primary atmospheric window from 8 *μ*m to 13 *μ*m, and around half in the secondary atmospheric window from 16 *μ*m to 22 *μ*m), as shown in Fig. 1. The primary window contributes ~90% of the total radiative cooling power at near-ambient temperature due to much higher transmittance than the second atmospheric window^6^. The absorptance of the PMP films rise within these two atmospheric transmission windows with the increasing thickness of PMP films. FTIR spectroscopy is executed to identify different molecular structures and compositions in samples through the transmission spectra or attenuated total reflection (ATR) mode^27,28^. Both methods can display the distinct absorption bands, however, ATR is often used to measure surface properties relying on the total reflectance of evanescent waves and has a penetration depth of 1 *μ*m or 2 *μ*m depending on the ATR crystal material^29^.

**Figure 2:**
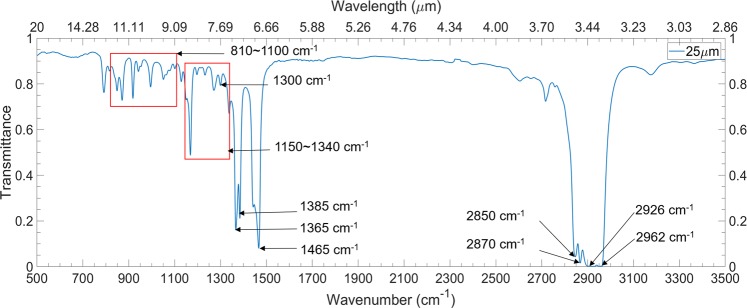
Transmittance measurement of a 20 *μ*m PMP free-standing film showing the various functional groups and their corresponding molecular vibration modes.

It is obvious that the transmission spectrum analysis method is more suitable for much thicker PMP films. Distinct absorption peaks are clearly visible in PMP film transmittance spectra when the thickness is less than 50 *μ*m since the absorption peaks merge as shown in the transmission spectra of thicker PMP films, such as 457 *μ*m or 1283 *μ*m thick samples in Fig. 1. While the thinner films show clearly distinct absorption peaks, thicker films display broadband absorptions, however, they cannot illustrate all the necessary functional groups or chemical bonds of PMP. It is reasonable that the transmittance spectrum of the 20 *μ*m film (Fig. 2) is selected to analyze the relationship between highly absorptance atmospheric windows and the chemical bonds of PMP. Evidently, there exist two different absorptions peaks: strong absorption peaks lie around 1400 cm^−1^ and 2900 cm^−1^, and weak absorption peaks lie in 810 cm^−1^~1100 cm^−1^ and 1150 ~ 1340 cm^−1^. The most notable absorption peaks around 2900 cm^−1^ are owing to the symmetric or asymmetric stretch vibrations of CH_2_ and CH_3_: asymmetric stretch vibrations of CH_2_ and CH_3_ bonds at 2850 cm^−1^ (3.51 *μ*m) and 2962 cm^−1^ (3.38 *μ*m), respectively; symmetric stretch vibrations of CH_2_ and CH_3_ at 2926 cm^−1^ (3.42 *μ*m) and 2870 cm^−1^ (3.48 *μ*m), respectively. The strong absorption peak at 1465 cm^−1^ (6.83 *μ*m) denotes the asymmetric scissoring bending vibrations of CH_3_ bond. Normally, the symmetric scissoring bending vibrations of CH_3_ bond are located at 1375 ± 5 cm^−1^ (7.26 *μ*m)^30^. However, when two or three CH_3_ groups are attached to the same carbon atom, the symmetrical scissoring bending vibration of CH_3_ will couple and cause the absorption band to split into two distinct peaks. For PMP, there are two CH_3_ groups that are connected to one carbon atom, so the symmetrical scissoring bending vibration of CH_3_ divides into two separated absorption peaks: 1365 cm^−1^ (7.32 *μ*m) and 1385 cm^−1^ (7.22 *μ*m). Since these absorption peaks resulting from strong bonds vibrations, the absorptance approaches to unity even though the thickness of PMP films is small. However, the weak absorption peaks are situated at 810 cm^−1^ ~ 1100 cm^−1^ (9.09 *μ*m~12.34 *μ*m) and 1150 cm^−1^~1340 cm^−1^ (7.46 *μ*m~8.69 *μ*m). The corresponding absorption peaks increase with the thickness of PMP films. The CH_3_ wagging bending vibrations and CH_2_ out-of-plane wagging bending vibrations illustrate the weak absorptions peaks at 810 cm^−1^~1100 cm^−1^ and 1150 cm^−1^~1340 cm^−1^, respectively. These absorption peaks in the 20 *μ*m PMP film are attributed to surface phonon modes^31^ corresponding to different vibration modes.

### Refitting of complex refractive indices

Complex refractive indices of PMP are extracted by refitting measured transmittance spectra to calculated transmittance data. Six samples (thickness: 20 *μ*m, 48 *μ*m, 144 *μ*m, 199 *μ*m, 386 *μ*m, and 1283 *μ*m) of PMP films are chosen to extract the complex refractive indices, which are used to calculate transmission spectra of other two samples of thickness 100 *μ*m and 457 *μ*m for the validation of the extracted refractive indices of PMP films.

Lorentz-Drude oscillator model for dielectric function of PMP is employed to calculate the refitted transmittance spectrum, which is given by^32^

1 $$\varepsilon (\omega )={\varepsilon }_{\infty }+\mathop{\sum }\limits_{k\mathrm{=1}}^{N}\,\frac{{s}_{k}}{1-{(\frac{\omega }{{\omega }_{k}})}^{2}-j{\Gamma }_{k}(\frac{\omega }{{\omega }_{k}})}$$

Here, *s*_*k*_, *ω*_*k*_, Γ_*k*_ and *j* are the strength, resonant frequency, damping factor of *k* th Lorentz-Drude oscillator and the imaginary unit, respectively. *N* such oscillators are assumed. *ε*_*∞*_ is the contribution from higher frequencies. The complex refractive indices $\sqrt{\varepsilon \mu }=n+j\kappa$, where *ε* and *μ* are the permittivity and permeability, respectively. *n* and *κ* are the refractive index and extinction coefficient, respectively. Since PMP is a non-magnetic polymer, its permeability *μ* is 1. The generalized transmissivity is given by

2 $$\tau =\frac{1}{2}[|{T}^{TE}{|}^{2}+|{T}^{TM}{|}^{2}]$$

where *T*^*TE*^ and *T*^*TM*^ are effective transmission coefficients at the given angle of incidence for transverse electric (TE) and transverse magnetic (TM) polarization, respectively. In this work, a thin film structure of air-PMP-air is considered for the normal transmission calculation and measurement. The generalized transmission coefficient for the interface between air and PMP can be expressed as^33^

3 $${T}^{(\beta )}=\frac{{T}_{\mathrm{1,2}}^{(\beta )}{e}^{j{k}_{1z}d}}{1-{R}_{\mathrm{1,2}}^{(\beta )}{\tilde{R}}_{\mathrm{2,3}}^{(\beta )}{e}^{2j{k}_{2z}d}}$$

where *T*_1,2_^(*β*)^ and *R*_1,2_^(*β*)^ are the Fresnel transmission and reflection coefficient at the interface between layer 1 (air) and layer 2 (PMP film), respectively. ${\tilde{R}}_{\mathrm{2,3}}^{(\beta )}$ is the Fresnel reflection coefficient at the interface between layer 2 (PMP) and layer 3 (air). *β* = *s* (or *p*) is for TE (or TM) polarization, *d* is the thickness of PMP film. ${k}_{iz}=\sqrt{{\varepsilon }_{i}(\omega ){\omega }^{2}/{c}^{2}-{k}_{\rho }^{2}}$ is the normal component of the wave vector in air and PMP film*, ε*_*i*_(*ω*) is the permittivity of air (*i* = 1*, ε*_1_(*ω*) = 1) and PMP (*i* = 2) as a function of angular frequency *ω*, *c* is the speed of light in the vacuum and *k*_*ρ*_ = sin(*θ*)*ω*/*c. θ* is the incident angle of beam to PMP films. For this case, the incident angle for transmission measurement is 0°.

Dielectric function of PMP can be extracted by tuning several oscillator parameters in order to match the measured transmittance spectra of these six samples. An optimization procedure is used to minimize the error between measured and refitted spectra. The error between these two spectra is given by

4 $$\begin{array}{rcl}\delta  & = & {\mathop{\sum }\limits_{i\mathrm{=1}}^{M}{[{T}_{m}-{T}_{r}]}^{2}|}_{20\mu m}+{\mathop{\sum }\limits_{i\mathrm{=1}}^{M}{[{T}_{m}-{T}_{r}]}^{2}|}_{48\mu m}+{\mathop{\sum }\limits_{i\mathrm{=1}}^{M}{[{T}_{m}-{T}_{r}]}^{2}|}_{144\mu m}\\  &  & {+{\mathop{\sum }\limits_{i\mathrm{=1}}^{M}{[{T}_{m}-{T}_{r}]}^{2}|}_{199\mu m}+{\mathop{\sum }\limits_{i\mathrm{=1}}^{M}{[{T}_{m}-{T}_{r}]}^{2}|}_{386\mu m}+\mathop{\sum }\limits_{i\mathrm{=1}}^{M}{[{T}_{m}-{T}_{r}]}^{2}|}_{1283\mu m}\end{array}$$

**Figure 3:**
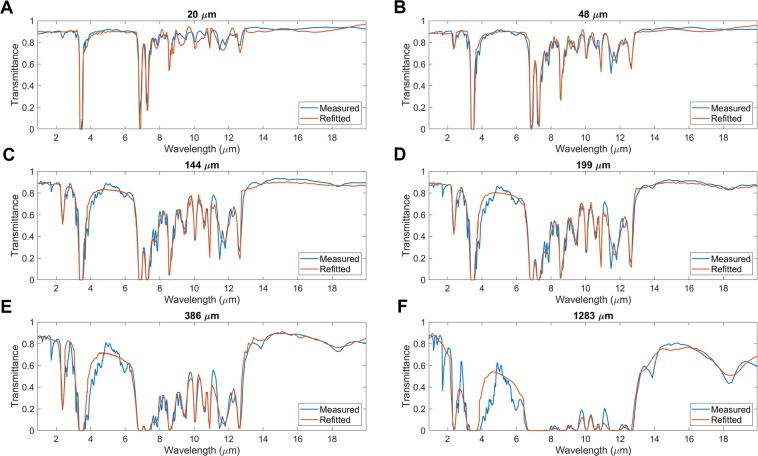
Transmittance measurement of PMP free-standing films of various thicknesses, (**A**) 20 *μ*m, (**B**) 48 *μ*m, (**C**) 144 *μ*m, (**D**) 199 *μ*m, (**E**) 386 *μ*m, and (**F**) 1283 *μ*m, in comparison with refitted spectra using the Lorentz-Drude oscillator model.

**Figure 4:**
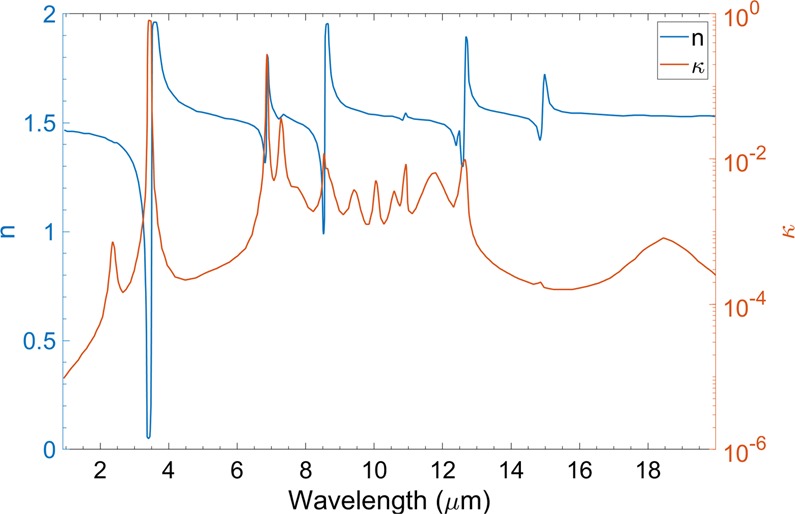
Real (*n*) and imaginary (*κ*) parts of the complex refractive indices of PMP extracted by refitting the transmittance spectra.

Here, *T*_*m*_ and *T*_*r*_ are measured and refitted values of transmittance, respectively. Index *i* indicates different wavelengths over which the transmittance measurements are conducted. MATLAB based genetic algorithm is applied to obtain the final optimized resonant frequencies and achieve a good fit between measured and refitted spectra. It can be empirically found that 21 groups of oscillator parameters are suitable to approach the best fit. Comparisons of measured and refitted transmittance spectra are shown in Fig. 3A (20 *μ*m), 3B (48 *μ*m), 3C (144 *μ*m), 3D (199 *μ*m), 3E (386 *μ*m), and 3F (1283 *μ*m), which display good fits for all six PMP samples. Most of the absorption peaks for various PMP films are fitted using the oscillator parameters listed in Table 1. The deviations between the measured and refitted spectrum increases as the thickness increases, which can be seen in Fig. 3F. This is because there are fewer absorption peaks in the sample of 1283 *μ*m compared with other samples, and it is hard to get a good fit using a set of same oscillator parameters. The refitting method optimizes these oscillator parameters which is corresponding to these absorption peaks to get a good fit between measured and refitted spectra. The real and imaginary parts of the complex refractive indices of PMP are shown in Fig. 4. The higher extinction coefficients (*κ*) around 3.4 *μ*m (2900 cm^−1^) and 6.8 *μ*m (1470 cm^−1^) result from the strong stretch and bending vibrations of CH_2_ and CH_3_ bonds. The weak wagging bending vibrations of CH_2_ and CH_3_ bonds denote appearances of small peaks at extinction coefficients between 7.22 *μ*m to 12.34 *μ*m, which corresponds to the primary atmospheric window.

**Table 1:** Oscillator parameters of PMP used to refit the transmittance spectra.

k	*ω*_*k*_	*λ*_*k*_	*s*_*k*_	Γ_*k*_
—	(cm^−1^)	(*μ*m)	—	—
		*ε*_∞_ = 1.4		
1	5415	1.84	9.907E-08	0.999E-00
2	4197	2.38	6.149E-05	2.721E-02
3	3968	2.52	2.153E-07	0.999E-00
4	4050	2.47	2.655E-04	2.935E-06
5	2863	3.49	7.570E-02	1.822E-04
6	1818	5.50	1.756E-04	3.010E-01
7	1456	6.87	4.936E-03	1.082E-03
8	1372	7.28	8.621E-04	7.413E-03
9	1290	7.75	3.161E-04	3.394E-02
10	1166	8.58	7.217E-03	3.727E-07
11	1163	8.60	3.532E-03	3.734E-05
12	1153	8.67	3.227E-04	1.968E-02
13	1061	9.42	1.638E-04	1.635E-02
14	995	10.05	8.696E-05	5.278E-03
15	946	10.57	9.078E-05	1.026E-02
16	919	10.88	1.880E-04	8.700E-04
17	853	11.72	5.537E-04	2.874E-02
18	804	12.44	7.769E-04	6.204E-05
19	792	12.62	4.886E-03	6.805E-05
20	670	14.91	2.036E-03	2.385E-07
21	542	18.45	1.105E-04	4.915E-02

### Validation of extracted refractive indices

**Figure 5:**
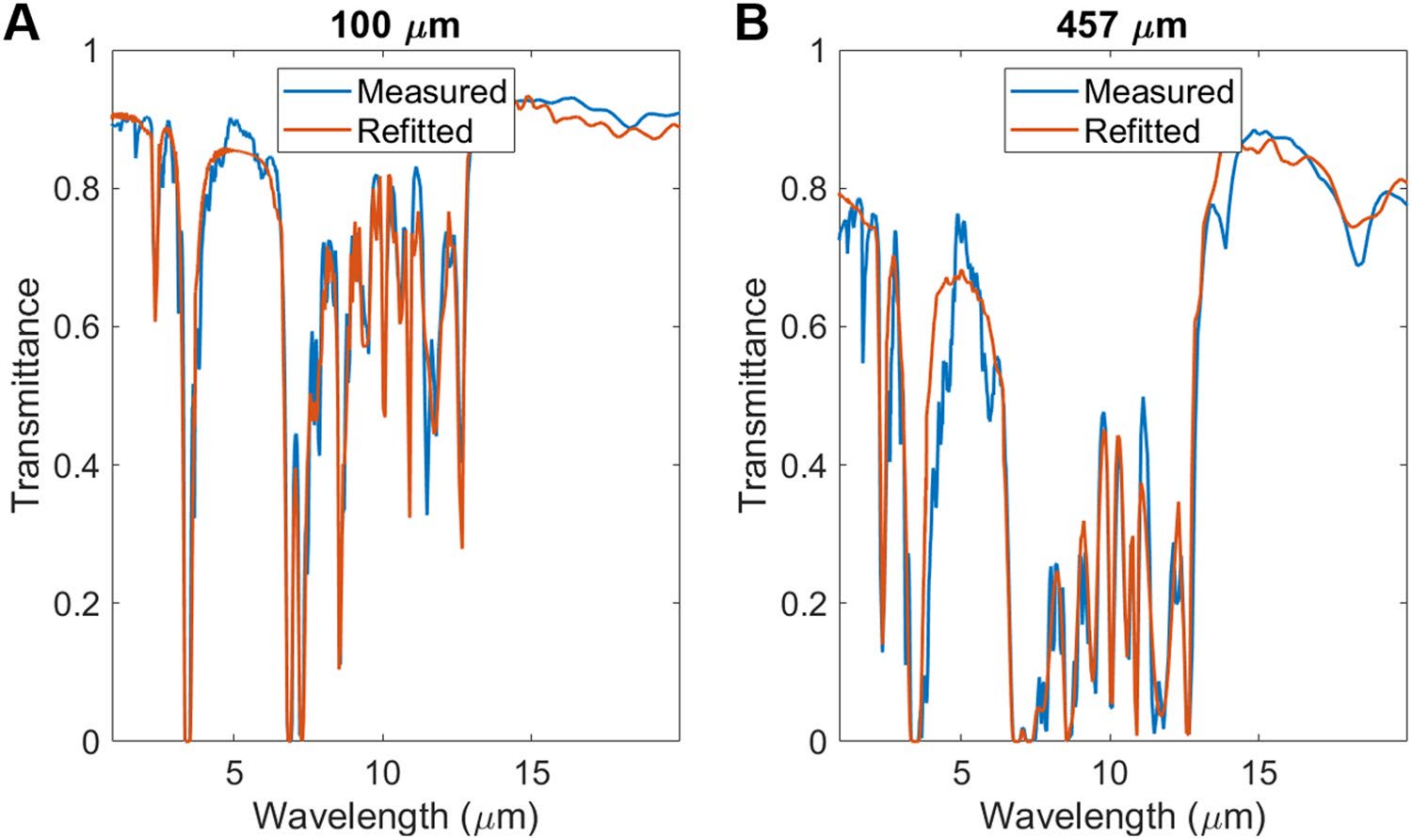
Validation of extracted complex refractive indices through comparisons between measured transmittance and refitted spectra for (**A**) 100 *μ*m and (**B**) 457 *μ*m thick PMP samples.

In order to validate the refitting procedure of PMP films, transmittance spectra are evaluated for 100 *μ*m and 457 *μ*m thick PMP samples using Eqs. (2) and (3) with the extracted complex refractive indices (Fig. 4). The measured and refitted transmittance spectra of PMP thin films with thicknesses of 100 *μ*m and 457 *μ*m show a well match as shown in Fig. 5A,B, respectively. These 12 main absorption peaks of these two curves occurs at the same wavelength. It confirms that the extracted refractive indices of PMP can be used to predict the optical properties of PMP related thermal photonic structures.

## Discussion

### PMP film based radiative cooler

**Figure 6:**
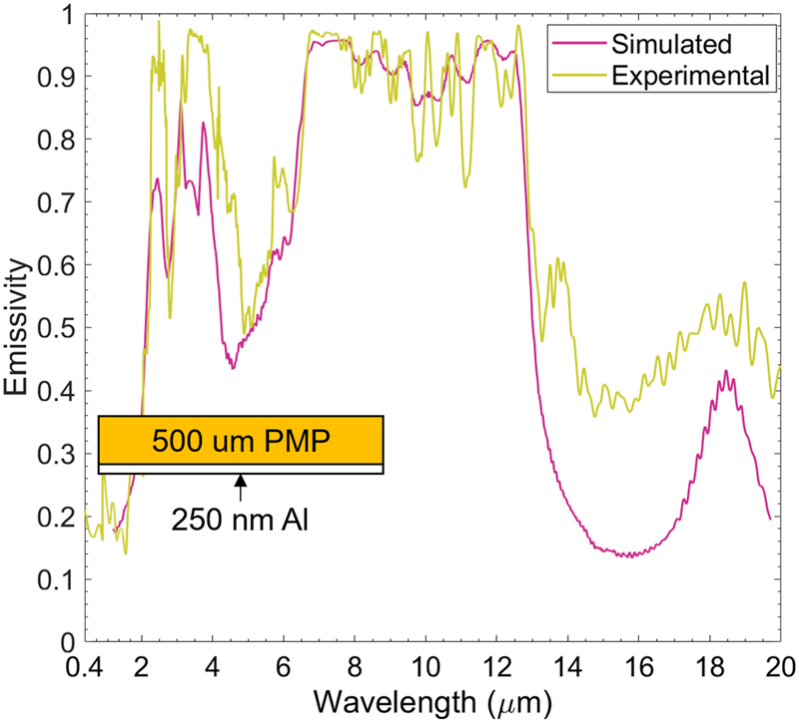
Measured and simulated emittance spectra of a 500 *μ*m PMP free standing film sputtered 250 nm thin aluminium on its backside.

Aluminized PMP films are easily manufactured structures serving as a radiative cooler. Magnetron sputtering technology is employed to deposit a 250 nm thin aluminum on the back of a 500 *μ*m PMP film, which is thick enough to block all the lights from visible to mid-infrared range^34^. The diffused FTIR reflectance measurements follow the procedures to be discussed in the method section. The emissivity of the radiative cooler of interest can be obtained from the reflectance measurement based on Kirchhoff’s law by *ε* = 1 − *τ* − *ρ*, here *ρ* is the measured reflectance and transmittance *τ* is zero. As shown in Fig. 6, the simulated emissivity curve of the sample matches well with the experimental result and it shows low emissivity in the solar radiation wavelength region and high emissivity in the two earth atmospheric transparency windows (highlighted regions in blue). Since the refitting method optimizes these oscillator parameters which correspond to these absorption peaks to get a good fit between measured and refitted spectra, the discrepancy arises outside the primary atmospheric window, where most of these absorption peaks locates.

### Thermal performance investigation

In order to demonstrate the photon-to-cooling capability of the radiative cooler under direct solar irradiation, its temperature variations are simulated by solving the thermal balance equation as expressed by^35,36^

5 $${Q}_{total}={Q}_{cooler}({T}_{cooler})-{Q}_{conv}({T}_{amb},{T}_{cooler})-{Q}_{amb}({T}_{amb})-{Q}_{sun}({T}_{cooler})$$

It is supposed that the backside of the PMP cooler is insulated, we just consider the heat transfer between PMP cooler, air and outer space. Here, *Q*_*cooler*_ is the net cooling power of the radiative cooler, *Q*_*conv*_ is due to the natural air convective heat transfer, *Q*_*amb*_ is the incident thermal radiation from ambient, and *Q*_*sun*_ stands for the incident solar irradiation absorbed by the radiative cooler. *T*_*cooler*_ and *T*_*amb*_ are the temperatures of radiative cooler and ambient air, respectively. *Q*_*cooler*_ can be determined as follows:

6 $${Q}_{cooler}({T}_{cooler})=A{\int }_{0}^{\infty }d\lambda {I}_{BB}({T}_{cooler},\lambda )\varepsilon (\lambda ,\theta ,\phi ,{T}_{cooler})$$

where, *A* is the area of radiative cooler, *I*_*BB*_(*T*_*cooler*_,*λ*) = 2*hc*^2^*λ*^−5^ *exp*(*hc*/*λk*_*B*_*T*−1)^−1^ defines the spectral radiance of blackbody at a certain temperature *T*, where *h* is the Planck’s constant, *k*_*B*_ is the Boltzmann constant, and *λ* is the wavelength. *ε*(*λ*, *θ*, *ϕ*, *T*_*cooler*_) = $$\frac{1}{\pi }$$$${\int }_{0}^{2\pi }{\rm{d}}\phi {\int }_{0}^{\pi \mathrm{/2}}{\varepsilon }_{\lambda }\cos \,\theta \,\sin \,\theta {\rm{d}}\theta $$ is the temperature-dependent emissivity of radiative cooler^37^. Here, the emissivity measured at room temperature (298 K) is taken into simulation, since it is assumed that the temperature variations of PMP and aluminum affect little on emissivity measurement*. θ* and *ϕ* are the azimuthal and latitudinal angles, respectively.

The parasitic heat transfer between the radiative cooler and ambient air is given by

7 $${Q}_{conv}({T}_{amb},{T}_{cooler})=A{h}_{a}({T}_{amb}-{T}_{cooler})$$

*h*_*a*_ is the nonradiative heat transfer coefficient ranging from 2 to 8 Wm^−2^ K^−1^ ^8,35,38^. Here *h*_*a*_ = 8 Wm^−2^ K^−1^ is set as natural air conduction and convection heat transfer to the radiative cooler. The absorbed power from incident thermal radiation from atmosphere *Q*_*amb*_(*T*_*amb*_) is given by

8 $${Q}_{amb}({T}_{amb})=A{\int }_{0}^{\infty }{\rm{d}}\lambda {I}_{BB}({T}_{amb},\lambda )\varepsilon (\lambda ,\theta ,\phi ,{T}_{cooler})\varepsilon (\lambda ,\theta ,\phi )$$

The absorptivity of the atmosphere, *ε*(*λ*,*θ*,*ϕ*), is given by 1 − *τ*(*λ*,*θ*,*ϕ*). Here *τ*(*λ*,*θ*,*ϕ*) is the transmittance value of atmosphere obtained from MODTRAN4^39^. Solar irradiation absorbed by the radiative cooler *Q*_*sun*_(*T*_*cooler*_) is given by

9 $${Q}_{sun}({T}_{cooler})=A{\int }_{0}^{\infty }{\rm{d}}\lambda {I}_{AM1.5}(\lambda )\varepsilon (\lambda ,{\theta }_{sun},{T}_{cooler})$$

Here, *I*_AM1.5_(*λ*) is the spectral irradiance intensity of solar irradiation at AM 1.5. *ε*(*λ*, *θ*_*sun*_, *T*_*cooler*_) is the temperature-dependent emissivity of radiative cooler, we take the measured data of the radiative cooler at room temperature into consideration.

The time-dependent temperature variations of the radiative cooler can be obtained by solving the following equation

10 $${C}_{cooler}\frac{dT}{dt}={Q}_{total}({T}_{cooler},{T}_{amb})$$

Since the aluminum is sputtered on the PMP film, thermal contact resistance is negligible. The heat capacitance of the radiative cooler, *C*_*cooler*_, consists of PMP (500 *μ*m) and aluminum (250 nm).

**Figure 7:**
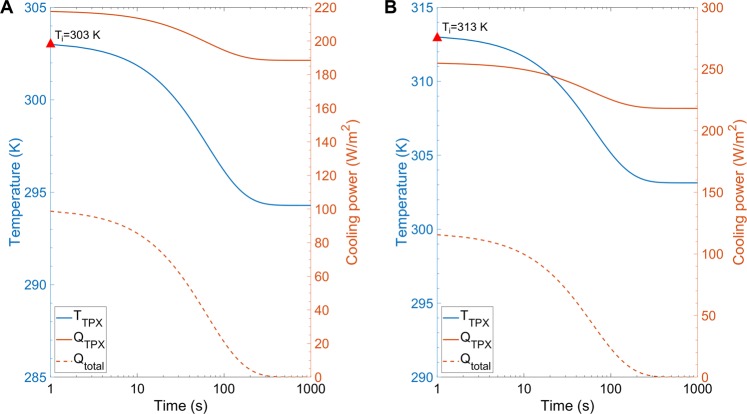
Transient thermal performance of the radiative cooler at different initial temperatures assumed to be the same as ambient temperature. Time-dependent evolution of *T*_*PMP*_, *Q*_*PMP*_ and *Q*_*total*_ at (**A**) 303 K and (**B**) 313 K ambient temperature.

The transient temperature fluctuations of the radiative cooling system under two different ambient temperature conditions are simulated by solving Eq. (10), which is integrated to obtain the temperature evolution of radiative cooler as a function of time, as shown in Fig. 7. For each simulation, the initial temperature of the radiative cooler (*T*_*i*_) is assumed to be the same as the ambient temperature.

When the ambient temperature is 303 K, the temperature of the radiative cooler, *T*_*PMP*_, reduces and eventually reaches a thermal equilibrium temperature that is about 9 K below the ambient temperature because of the cooling effects of radiative cooler (blue solid curve in Fig. 7A). The photon-to-cooling performance of the radiative cooler *Q*_*PMP*_ remains over 180 W/m^2^ and reduces slightly with the evolution of time (orange solid curve in Fig. 7A). The net output of the radiative cooler *Q*_*total*_ reduces and approaches zero when the cooler is at thermal equilibrium condition (orange dashed curve in Fig. 7A). Similarly, when the ambient temperature is 313 K, as shown in Fig. 7B, the temperature of the radiative cooler eventually drops around 10 K below the ambient temperature. The cooling power *Q*_*PMP*_ and net output *Q*_*total*_ of the cooler follow the same behaviors as described in the previous case of the ambient temperature of 303 K.

**Figure 8:**
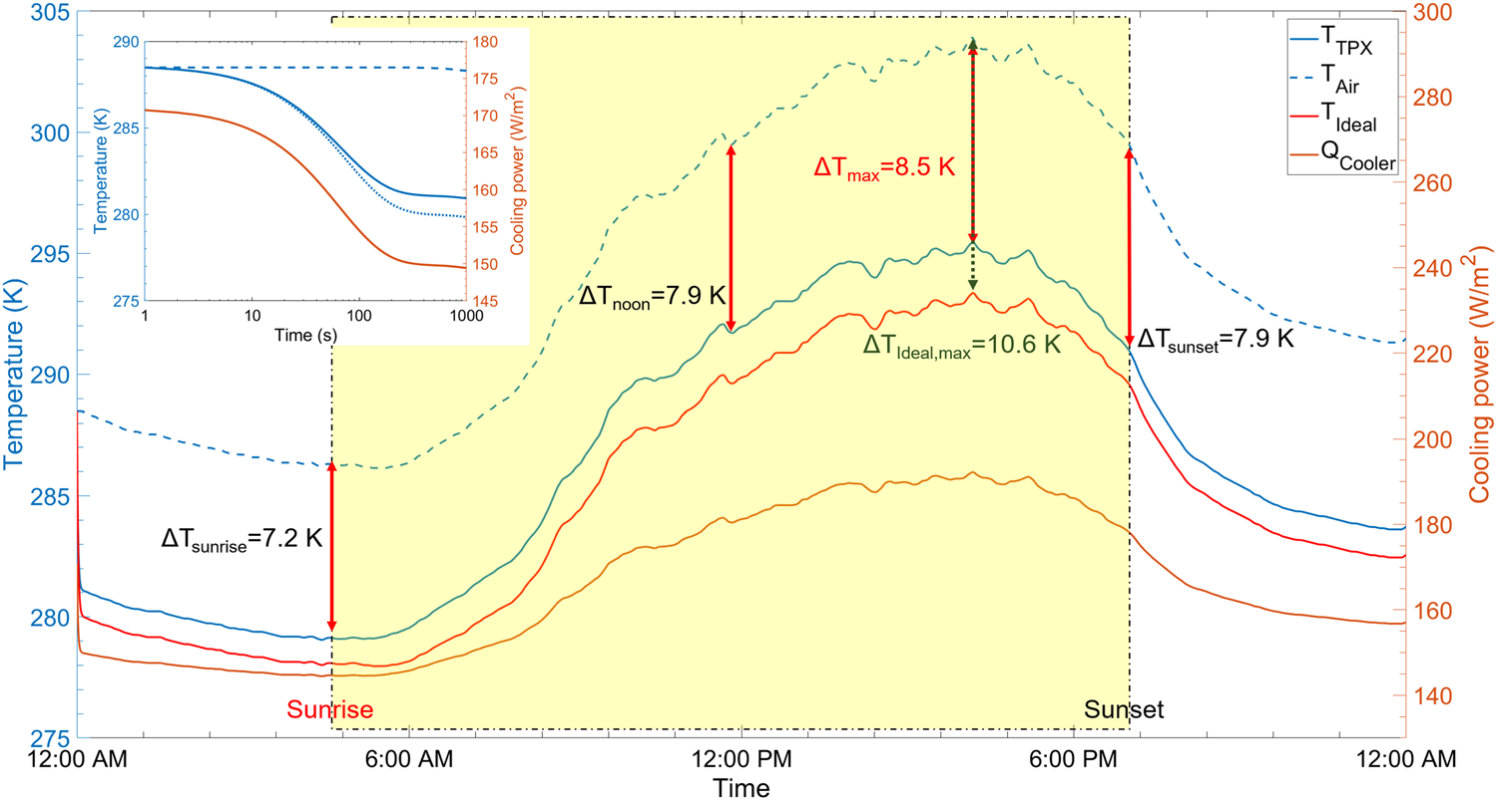
Left *y*-axis: thermal performance of the actual radiative cooler (blue solid curve) and the ideal one (blue dotted curve) over a 24-hour cycle at varying ambient temperatures (blue dashed curve). Right *y*-axis: net radiative cooling power (orange solid curve). Inset: Time-dependent evolution of thermal performance of a radiative cooler for the very beginning 1000 seconds.

In the end, the radiative cooling temperature response is simulated under a real weather condition on July 20, 2018, in Santa Clare, California^40^, which has a typical summer climate. Using the ambient temperature and solar illumination data of July 20, 2018^41^, as inputs of Eq. (5), the net radiative cooling power of radiative cooler and temperatures are simulated over a 24-hour period, as shown in Fig. 8. The ideal radiative cooler is set to be with unity emissivity in the atmospheric transparency window while it has zero emissivity at other wavelengths, and it yields a maximum temperature drop ΔT of 10.6 K. The inset of Fig. 8 displays the temperature difference between the radiative cooler and the ambient occurs and reaches thermal equilibrium in about 300 s, then it fluctuates with the variations of ambient temperature. The temperature drops ΔT at sunrise, noon, and sunset remain over 7 K. It promises sufficient cooling effects. The maximum temperature difference of about 8.5 K occurs at around 3:30 PM when both the net radiative cooling power and the ambient temperature reach to the maximum^42,43^.

## Methods

### Sample preparation and instruments

**Figure 9:**
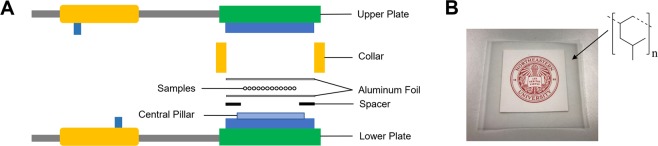
(**A**) Schematic of hot-press mold for thin film preparation. (**B**) The Northeastern University logo covered by a 76 *μ*m thick PMP film and the wireframe of PMP.

PMP granules with a nominal size of 3 mm are purchased from GoodFellow, Inc. (USA) and sodium hydroxide (NaOH) with a purity of 98% is supplied by Sigma-Aldrich Co. Inc. (USA). The electric auto hydraulic duel rosin press machine with PID temperature control of 1 °C accuracy is purchased from TechTongDa, Inc. (USA). The hot-press mold (Fig. 9A) is home built, and the spacer has an inner diameter of 1 inch and an outer diameter of 1.5 inches with various thicknesses. The aluminum target of 99.999% purity is purchased from Angstrom Sciences. The PMP sheet with a thickness of 500 *μ*m is purchased from CS Hyde Company (USA). The thickness of PMP thin films is measured by iGaging digital electronic micrometer with an accuracy of ±4 *μ*m.

### PMP films preparations for transmittance measurement

First, turn on the hydraulic press machine and preheat the pressing plate above the melting temperature of PMP (250 °C), considering the heat resistance between different parts of the hot-press mold. All the parts of the hot-press mold are cleaned using successive washes of acetone and deionized water and dried with dry nitrogen gas, then put the lower platen, collar, the bottom aluminum foil, and spacer successively. A certain amount of PMP granules are put on the bottom aluminum foil at the center of the spacer according to the desired thickness and density of PMP films. After PMP granules melt, carefully put the top aluminum foil and the upper plate, then start to press with a pressure of 70 psi. 1 minute later, take out the hot-press mold and cool down to the room temperature. For the sake of obtaining intact films, put the PMP thin film with aluminum foils into 3 mol/L NaOH solution until the aluminum foils dissolve. Finally, take the PMP thin film out from NaOH solution and wash with deionized water, then put PMP thin film into a preheated 60 °C convection oven for 20 minutes to release adsorbed water before transmittance spectra measurement. Through changing the amount of PMP granules and the thickness of spacers, PMP thin films of various thicknesses can be obtained, a PMP thin film with a thickness of 76 *μ*m is shown in Fig. 9B.

### Aluminized PMP radiative cooler preparations

A 250 nm thin aluminum film is deposited on one side of the PMP sheet via a home-built high vacuum magnetron sputtering machine. The base pressure (vacuum before sputtering starts) and Argon pressure during sputtering are 4.2 × 10^−4^ mTorr and 0.87 mTorr, respectively. The aluminum thin film is deposited on the PMP sheet with a sputtering rate of 0.0838 nm/s at 15 W DC power supply.

## Transmittance and Reflectance Measurement

### Transmittance measurement

**Figure 10:**
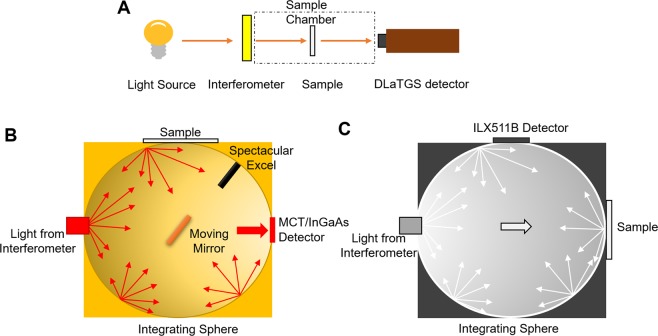
(**A**) Schematic of an experimental setup of the transmittance measurement. (**B**) Schematic of diffuse reflectance measurement accessory with an integrating sphere for near-infrared (0.9 *μ*m~2.5 *μ*m) and mid-infrared (2.5 *μ*m~20 *μ*m) reflectance measurement. (**C**) Schematic of diffuse reflectance measurement accessory with an integrating sphere for Visible (0.38 *μ*m~0.74 *μ*m) and near-infrared (0.74 *μ*m~0.85 *μ*m) region measurements.

The transmittance spectra of PMP thin films are measured with the experimental setup shown in Fig. 10A. The light source, interferometer, and Deuterated Lanthanum *α* Alanine doped TriGlycine Sulphate (DLaTGS) detector are located inside the Jasco 6600 Fourier Transform Infrared Spectroscopy (FTIR) spectrometer chamber. Jasco 6600 are equipped with halogen light source (from 0.9 *μ*m to 2.5 *μ*m) and ceramic light source (from 2.5 *μ*m to 25 *μ*m). The DLaTGS detector has a high accuracy from 0.9 *μ*m to 20 *μ*m. The samples of PMP thin films are sandwiched between infrared sample cards with an aperture of 17 mm. These samples are placed vertically to the optical path inside the sample chamber with a Jasco infrared cards holder. Each transmittance measurement includes a background measurement and a sample measurement. Scan rate is set to 32 with a resolution of 4 cm^−1^. Three measurements are taken for each sample and the average value of three measurements is taken as the final result for further analysis.

### Diffuse reflectance measurement

Diffuse reflectance measurements of near-infrared and mid-infrared are characterized using FTIR spectrometer equipped with PIKE’s diffuse reflectance accessory equipped with an integrating sphere, as shown in Fig. 10B. The accessory has a spherical shell with an inner surface coated with a diffuse reflective layer of gold and two infrared light detectors - indium gallium arsenide (InGaAs) (from 0.9 *μ*m to 2.5 *μ*m) and mercury cadmium telluride (MCT) detector (from 2.5 *μ*m to 20 *μ*m and cooled by liquid nitrogen). The integrating sphere has three ports: one is for light from the interferometer and the other two are for the sample and MCT/InGaAs detector, respectively. The infrared light from the interferometer falls on the moving mirror. When the moving mirror is turned to face to diffuse gold, the spectrometer takes the reference measurement (background reflectance is assumed to be 100%). The moving mirror can be switched to point at the sample with an incident angle fixed at 12°. The light reflected from the sample scatters at the inner surface of the integrating sphere and is captured by MCT/InGaAs detector. The scan rate is set to 64 with a resolution of 4 cm^−1^.

Diffused reflectance measurements of visible lights (0.38 *μ*m~0.74 *μ*m) and near-infrared lights (0.74 *μ*m~0.85 *μ*m) are characterized with Ocean Optics USB20000+ VIS-NIR-ES spectrometer equipped with a THORLABS 2-inch integrating sphere, as shown in Fig. 10C. This integrating sphere is inner-coated with a 1 mm thick PTFE based composite that has nearly unity reflectively within 0.25 *μ*m~2.5 *μ*m wavelength range. The integrating sphere has three ports: one is for light from the Ocean Optics halogen light source HL-2000 and the other two are for the sample/reflectance reference and Sony ILX511B detector that covers the wavelength from 0.35 *μ*m~1.0 *μ*m, respectively. First, take the background spectrum using the end-cap with a coating of 1 mm thick reflective material on the port, then place samples at the same port for measuring the reflectance spectrum. For these diffuse reflectance measurement discussed above, three measurements are taken from three different locations on each sample, and the averaged data is considered as the final results for further analysis.
